# FeV1 and BMI influence King’s Sarcoidosis Questionnaire score in sarcoidosis patients

**DOI:** 10.1186/s12890-021-01761-7

**Published:** 2021-12-03

**Authors:** Björn Christian Frye, Laura Potasso, Erik Farin-Glattacker, Surrinder Birring, Joachim Müller-Quernheim, Jonas Christian Schupp

**Affiliations:** 1 Department of Pneumology, Faculty of Medicine, Medical Center-University of Freiburg, University of Freiburg, Killianstrasse 5, 79106 Freiburg, Germany; 2 Department of Endocrinology, Diabetology and Metabolism, University Hospital Basel, Basel, Switzerland; 3 Section of Health Care Research and Rehabilitation Research, Faculty of Medicine,, Medical Center-University of Freiburg, Freiburg, Germany; 4 Division of Asthma, Allergy and Lung Biology, King’s College London, London, UK; 5 Section of Pulmonary, Critical Care and Sleep Medicine, Yale University School of Medicine, New Haven, CT USA

**Keywords:** Sarcoidosis, Quality of life, King’s Sarcoidosis Questionnaire

## Abstract

**Background:**

Sarcoidosis is granulomatous disease of unknown origin affecting organ function and quality of life. The King’s Sarcoidosis Questionnaire (KSQ) serves as a tool to assess quality of life in sarcoidosis patients with general health and organ specific domains. A German translation has been validated in a German cohort. In this study we assessed, whether clinical parameters influence KSQ scores.

**Methods:**

Clinical data (e.g. lung function, organ impairment, serological parameters) for the German validation cohort were extracted from clinical charts and investigated by correlation and linear regression analyses.

**Results:**

KSQ subdomain scores were generally lower in patients with respective organ manifestation or on current therapy. LUNG subdomain was significantly predicted by lung functional parameters, however for general health status, only FeV1 exerted significant influence. GHS was not influenced by serological parameters, but was significantly negatively correlated with body mass index (BMI). KSQ provides additional information beyond lung function, clinical or serological parameters in sarcoidosis patients. Notably, high BMI is significantly negatively associated with patients’ well-being as measured by KSQ-GHS.

**Conclusion:**

This observation may direct further studies investigating the effect of obesity on sarcoidosis-related quality of life and strategies to intervene with steroid-sparing therapies and measures of life style modifications.

*Trial registration* This study was registered in the German Clinical Trials Register (reference number DRKS00010072). Registered January 2016.

**Supplementary Information:**

The online version contains supplementary material available at 10.1186/s12890-021-01761-7.

## Background

Sarcoidosis is rare granulomatous disease that can affect virtually any organ [1, 2]. Besides measurable organ impairment (e.g. lung function defects) sarcoidosis affects quality of life (QoL) [3, 4] by organ-associated symptoms (e.g. cough) on the one hand and constitutional complaints (e.g. fatigue) on the other hand [1].

Several clinical trials in sarcoidosis have failed to meet primary endpoints based on improvement of measurable function impairment (e.g. forced vital capacity [FVC]) [5] even though patients experienced subjective improvement. Therefore, besides organ impairment, quality of life has been awarded an important endpoint for clinical studies in sarcoidosis [6]. To assess quality of life, Patel et al. presented a sarcoidosis-specific questionnaire [7] (King’s Sarcoidosis Questionnaire, KSQ) which has been translated and validated to Dutch and German [8, 9]. The questionnaire has 29 items covering general health status (GHS) as well as subdomains for lung (LUNG), skin (SKIN), eye (EYE), and medication (MED) associated QoL. Each subdomain can take a score between 0 and 100 and scores can be combined to integrate different subdomains in one score. In the initial analysis by Patel et al. [7], LUNG subdomain correlated well with lung function and the other subdomains correlated with respective organ involvement.

We have recently translated KSQ to German and validated its translated version in a cohort of sarcoidosis patients [8]. We hypothesized that KSQ offers additional information on patients’ well-being and that KSQ scores are only partially influenced by serological or lung function parameters routinely used to follow-up sarcoidosis patients.

## Methods

### Study cohort

The cohort was recently described in detail [8, 10]. Briefly, 200 consecutive sarcoidosis patients were recruited form the outpatient clinic of the Department of Pneumology after obtaining their informed consent. All patients completed KSQ independently and responded to demographic questions. Clinical data (including affected organs, lung function, body weight, size, soluble interleukin-2 receptor (sIL2R), neopterin and angiotensin-converting enzyme (ACE), radiological findings) were extracted from clinical charts. Lung functions were analyzed as percentage of the predicted normal value to allow analysis of all patients.

### Statistical analysis

Data were analyzed using *R* software version 4.0.0 (2020-04-24) [11]. We performed a full set analysis. Baseline characteristics were described as percentage of participants, mean ± standard deviation (SD) for normally distributed values or median and interquartile range (IQR), if values were not normally distributed.

To compare baseline characteristics of female and male patients, we performed a *Mann–Whitney* test in case of continuous variables, and a *chi square* test or a *Fisher’s exact* test in case of categorical variables. Box-plots were used to visualize differences between groups of participants, whereas we visualized continuous data results through scatter plots.

As a primary analysis, we computed a bivariate correlation analysis according to Pearson, in case of linear relationship, and a spearman rank test in case of no linearity. We then reported Pearson product-moment correlation coefficients with 95% confidence interval (CI) or Spearman’s rank rho coefficients with *p* values. In addition, we performed multivariable linear regression analyses with patients’ score as dependent variable and age, sex, BMI, lung function, radiological type (Scadding classification) affected organs, and sarcoidosis specific laboratory parameters as independent variables, in order to identify predictors for the scores. Variance inflation factor (VIF) analysis was computed to exclude multicollinearity of independent variables. A VIF value < 10 was considered acceptable [12]. Moreover, we performed uni- und multivariable linear regression analyses of KSQ subdomains (GHS, general health domain; LUNG, lung domain; EYE, eye domain; SKIN, skin domain; MED, medication domain), both as a sensitive analysis of the KSQ itself as well as to assess the influence of each subdomain on GHS. Domains can be merged, eg. general health status domain plus lung domain (GHS L) as shown in Table 1. To crossvalidate the multivariable models, we used k-fold cross-validation tests yielding in comparable results.

**Table 1:** Demographics of the study cohort

	Whole cohort		Female cohort (n = 97)		Male cohort (n = 103)		
	Median or Mean ± SD	IQR	Median or Mean ± SD	IQR	Median or Mean ± SD	IQR	p (female vs male)
Age	53.3 ± 12.7		55.0 ± 12.4		51.8 ± 12.8		*0.08*
BMI	28.0 ± 5.3		27.5 ± 4.9		28.4 ± 5.7		0.42
FVC	98.4	83.2–108.4	98.7	84.7–106.3	98.3	83.3–109.8	0.91
TLC	86.3	75.1–95.8	85.0	74.3–95.9	87.5	75.3–95.0	0.50
DLCOsb	80.2	69.9–90.9	80.4	71.3–89.4	78.8	67.9–91.2	0.80
FEV1	85.8	74.5–99.1	86.8	75.6–98.7	84.6	74.4–99.1	0.64
GHS	67.9	51.9–80.2	69.8	58.0–81.1	64.2	49.1–79.3	0.15
GHS L	73.0	56.7–82.2	74.2	59.1–84.0	68.0	53.4–80.9	0.28
GHS E	69.4	54.4–80.6	71.7	57.8–82.1	68.3	50.0–80.6	0.30
GHS S	72.1	60.0–83.6	75.4	63.6–85.0	67.9	55.7–80.7	*0.06*
GHS LS	73.1	59.7–83.7	77.4	62.3–85.4	70.8	50.8–81.6	0.15
GHS LM	72.5	59.2–82.0	76.8	62.2–83.2	69.4	54.6–81.9	0.14
GHS SM	72.3	61.6–82.7	76.3	67.6–84.4	68.8	57.8–79.2	**0.02**
GHS LSM	74.3	60.7–83.3	78.4	64.7–84.5	71.8	58.4–80.0	*0.06*
LUNG	77.8	58.3–93.1	76.4	58.3–93.1	77.8	58.3–93.1	0.88
MED	84.9	54.6–100	84.9	67.7–100	77.3	54.6–100	0.13
SKIN	88.2	70.6–100	94.1	70.6–100	82.4	70.6–100	0.15
EYES	77.0	52.7–93.2	77.0	53.4–93.2	73.0	52.7–93.2	0.63
Borg	0.5	0.0–2.0	0.5	0.0–2.0	0.5	0.0–2.8	0.50
sIL2R	513.5	380–713	506.5	378.8–679.5	528.5	380–735	0.59
Neopterin	11.5	8.4–16.4	12.2	9.0–16.3	10.3	7.7–16.5	*0.09*
ACE	35.4	22.7–48.9	39.1	25.6–51.2	28.8	20.6–43.8	*0.06*

Italics indicate values with borderline significance defined as 0.05

Values with statistical significance defined as *p* < 0.05 were indicated in bold

### Ethics

The study was approved by the local Ethic Committee of the University of Freiburg and the study was registered at the German Clinical Trial Register (DRKS00010072).

## Results

### Baseline characteristics

200 consecutive patients with sarcoidosis (mean age of 53 years and 51.5% male) of a recently described cohort [8, 10] were analyzed for this study (Table 1). ACE values were missing for 43 patients, among which clinical data were missing for 20 patients. These patients did not significantly differ from the overall cohort. Lung and lymph node involvement were the most frequent manifestations of sarcoidosis (Additional file 1: Table S1) with no gender preference in organ manifestation.

Male and females did not differ significantly in age, BMI lung function parameters and GHS score (Table 1 and Additional file 1: Fig. S1). However, KSQ values tend to be lower in male compared to female, which was statistically significant for the GHS SM (combined general health, skin, and medication score) module. Additionally, without statistical significance some serological parameters for sarcoidosis tended to be lower in males (Table 1).

#### KSQ demonstrates congruency with organ involvement

In the analyzed cohort, KSQ subdomains demonstrated a reasonable congruency between subdomain scores and respective organ involvement/medication (Additional file 1: Fig. S1 A–D). No difference was observed for the LUNG subdomain because most (88%) of included patients had lung affection. (Additional file 1: Table S1).

KSQ general health score (GHS strongly correlates with the different subdomains (adjusted R-score of the model 0.56, *p* < 0.001) emphasizing a good internal congruency. Each subdomain influences GHS in a univariable model (Additional file 1: Fig. S2, A-D) and in multivariable model LUNG subdomain was the most important subdmain for GHS (estimate 0.45; 95% CI 0.34–0.55, Additional file 1: Table S2) compared to the other subdomains (estimates between 0.11 und 0.16, 95% CI between 0.02 and 0.24, Additional file 1: Table S2). This signifies that, on average, a “1 point” increase in LUNG explained a “0.45 point” increase in GHS. *FeV1 and DLCO influence LUNG score.*

**Fig. 1:**
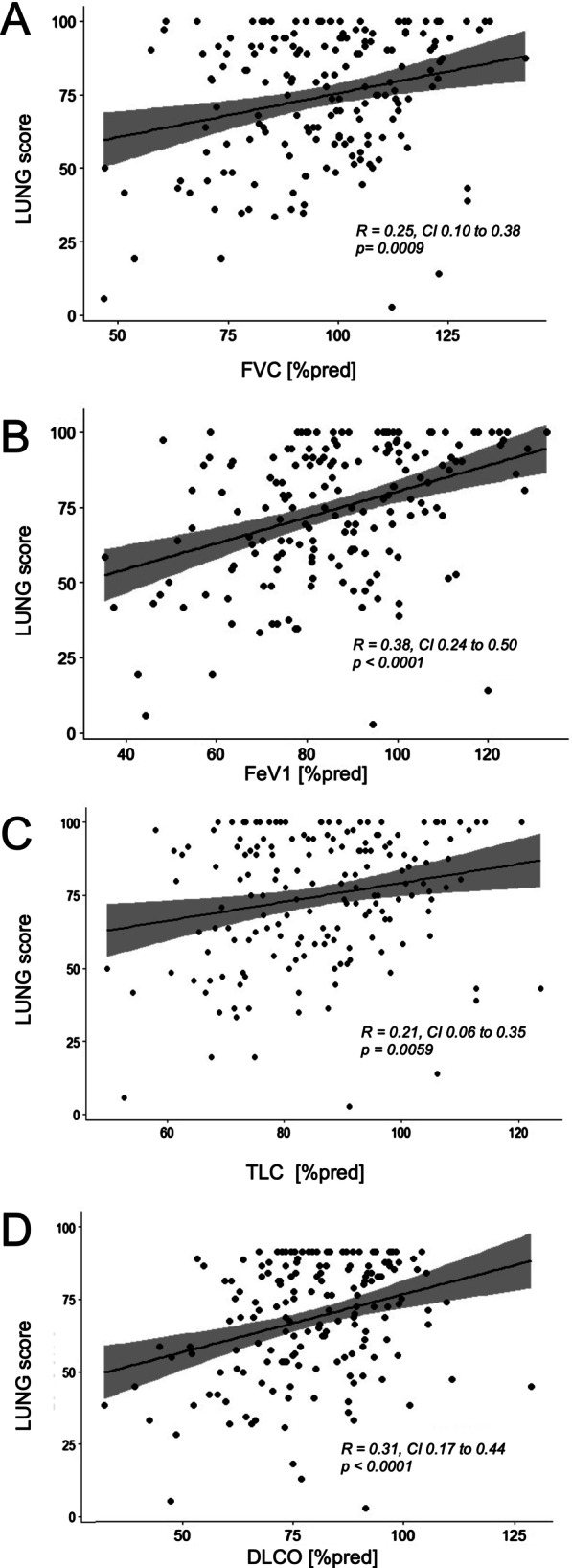
Correlation between lung function parameters and LUNG domain. Pearson’s correlations were calculated for different lung function parameters and LUNG score. **A** FVC positively correlated with LUNG score. **B** FeV1 positively correlated with LUNG score. **C** TLC positively correlated with LUNG score. **D** DLCO positively correlated with LUNG score

As lung is the most affected organ in sarcoidosis and LUNG score significantly effects GHS, we set out to further analyze to which extend clinical findings influence LUNG score. First, we assessed the correlation between lung function and LUNG score. Lung function parameters (especially FeV1) correlate with LUNG score with a slight gender difference (Fig. 1 and Table 2). A linear model confirmed these results and demonstrated that FeV1 and DLCO exerted the strongest influence on LUNG score (estimate 0.43, 95% CI 0.27–0.59 and estimate 0.53, 95% CI 0.13–0.71, *p* < 0.001 and *p* = 0.001 respectively, Additional file 1: Table S3), whereas the effects of FVC and TLC on LUNG score were weaker. Second, we used a multivariable linear model adding additional extrapulmonary organ involvement, age, sex, BMI, radiological type, and serological parameters used in sarcoidosis as independent variables to further elucidate, whether additional factors influence LUNG score. This model demonstrated a reasonable fitting (R^2^ of the model 0.41, *p* < 0.0001). As expected, extrapulmonary organ involvement had no influence on LUNG score (estimate -1.12, *p* = 0.80, Table 3) emphasizing the specificity of this subdomain score. Serological parameters used in sarcoidosis (ACE, neopterin and soluble interleukin-2 receptor) demonstrated no impact on LUNG score (Table 3), similar to sex. Of note, radiological classification according to Scadding did not correlate with LUNG score neither in this multivariable nor in univariable analyses, even though slight differences in lung function parameters between different Scadding classes could be detected (data not shown).

**Table 2:** Bivariate correlation for LUNG and lung function according to gender (spearman rank correlation)

	Female		Male	
	rho	*p*-value	rho	*p*-value
FVC	*0.21*	*0.08*	*0.20*	*0.05*
DLCO	**0.47**	**< 0.001**	*0.18*	*0.081*
TLC	0.12	0.336	**0.24**	**0.024**
FeV1	**0.36**	**0.002**	**0.39**	**< 0.001**

Italics indicate values with borderline significance defined as 0.05

Values with statistical significance defined as *p* < 0.05 were indicated in bold

**Table 3:** Multivariable model for LUNG score (adjusted R^2^ 0.33, *p* < 0.001)

	Estimate	CI	p-value	VIF
Intercept	50.16	5.48 to 94.84	0.028	na
Additional organ involvement	− 1.12	− 10.08 to 7.82	0.803	1.13
age	**0.36**	**0.08 to 0.64**	**0.012**	1.09
Female gender	− 5.95	− 13.24 to 1.34	0.109	1.17
**BMI**	− **1.09**	− **1.79 to **− **0.40**	**0.002**	1.26
**FEV1**	**0.70**	**0.33 to 1.07**	**0.0003**	4.42
FVC	− 0.33	− 0.79 to 0.13	0.154	5.85
**DLCOcSB**	**0.37**	**0.06 to 0.68**	**0.021**	1.95
TLC	− 0.26	− 0.67 to 0.15	0.210	2.99
Radio Type	− 1.75	− 6.13 to 2.63	0.429	1.38
Neopterin	0.46	− 0.21 to 1.25	0.179	1.41
ACE	− 0.002	− 0.04 to 0.03	0.922	1.20
sIL2-receptor	0.004	− 0.01 to 0.02	0.429	1.24

Values with statistical significance defined as *p* < 0.05 were indicated in bold

*VIF* Variance Inflation Factor

In this multivariable analysis, DLCO and FeV1 remained the only lung function parameters that significantly influenced LUNG score, whereas FVC and TLC did not (Table 3). Variance inflation factor (VIF) as a parameter for multicollinearity showed values below 10 for all parameters, indicating that multicollinearity is not a major problem, even though for FVC and FeV1 VIF is higher than for other parameters. When repeating the analysis without FVC, effect of FeV1, DLCO and BMI remain stable with lower VIF values (data not shown). On average, our patients showed an increase in LUNG score by 7.0 points if the predicted FeV1 meliorates by 10% (absolute increase). Interestingly, in addition to lung function parameters, body mass index (BMI) significantly lowers LUNG score by 1.09 points with every BMI gain of 1 kg/m^2^. In summary, these results demonstrate that LUNG score is only partially determined by lung function and therefore this score may provide additional information on disease burden beyond measurable parameters.

### Multiorgan involvement lowers GHS

KSQ is a quality of life questionnaire and general health perception is an important intent of this questionnaire. Sarcoidosis patients suffer from constitutional complaints that can often not be completely explained by organ manifestations, and underlying inflammation has been accused to cause these complaints.

As already mentioned, subdomains influence KSQ GHS (Additional file 1: Table S2). Using a multivariable analysis to assess the influence of different organ manifestations on GHS, we found that only bone involvement significantly lowered GHS score by 23 points (95% CI − 37.6 to − 8.5, *p* = 0.002, Table 4), whereas no other organ involvement has significant influence.

**Table 4:** Influence of organ manifestation, BMI, age and gender on GHS

	Estimate	CI	p	VIF
Eye involvement	− 8.490	− 19.15 to 2.17	0.118	1.16
Liver involvement	− 2.417	− 15.76 to 10.92	0.721	1.48
Lung involvement	− 4.078	− 13.58 to 5.42	0.398	1.08
Skin involvement	− 0.164	− 8.73 to 8.40	0.970	1.04
CNS involvement	2.077	− 9.07 to 13.23	0.713	1.11
Renal involvement	− 0.448	− 12.63 to 11.73	0.942	1.04
Heart involvement	− 4.250	− 21.20 to 12.70	0.621	1.04
Bone involvement	− **23.064**	− **37.63 to **− **8.49**	**0.002**	1.06
Joint	− 12.889	− 28.74 to 2.96	0.110	1.08
Spleen involvement	− 12.804	− 28.66 to 3.05	0.113	1.43
BMI	− **1.076**	− **1.61 to **− **0.54**	**0.0001**	1.05
Male	− 5.803	− 11.75 to 0.14	*0.056*	1.09
Age	− 0.004	− 0.23 to 0.22	0.969	1.04

Adjusted R^2^ = 0.17, *p* < 0.0001 for the model

Values with statistical significance defined as *p* < 0.05 were indicated in bold

*VIF* Variance Inflation Factor

The most likely explanation is that bone involvement is mainly found in patients with multiorgan sarcoidosis. It therefore may mirror multiple organs affected by sarcoidosis in these patients. In line with this hypothesis, multiple organ involvement significantly influenced GHS in our cohort (Additional file 1: Table S4).

### Higher BMI negatively influences GHS

**Fig. 2:**
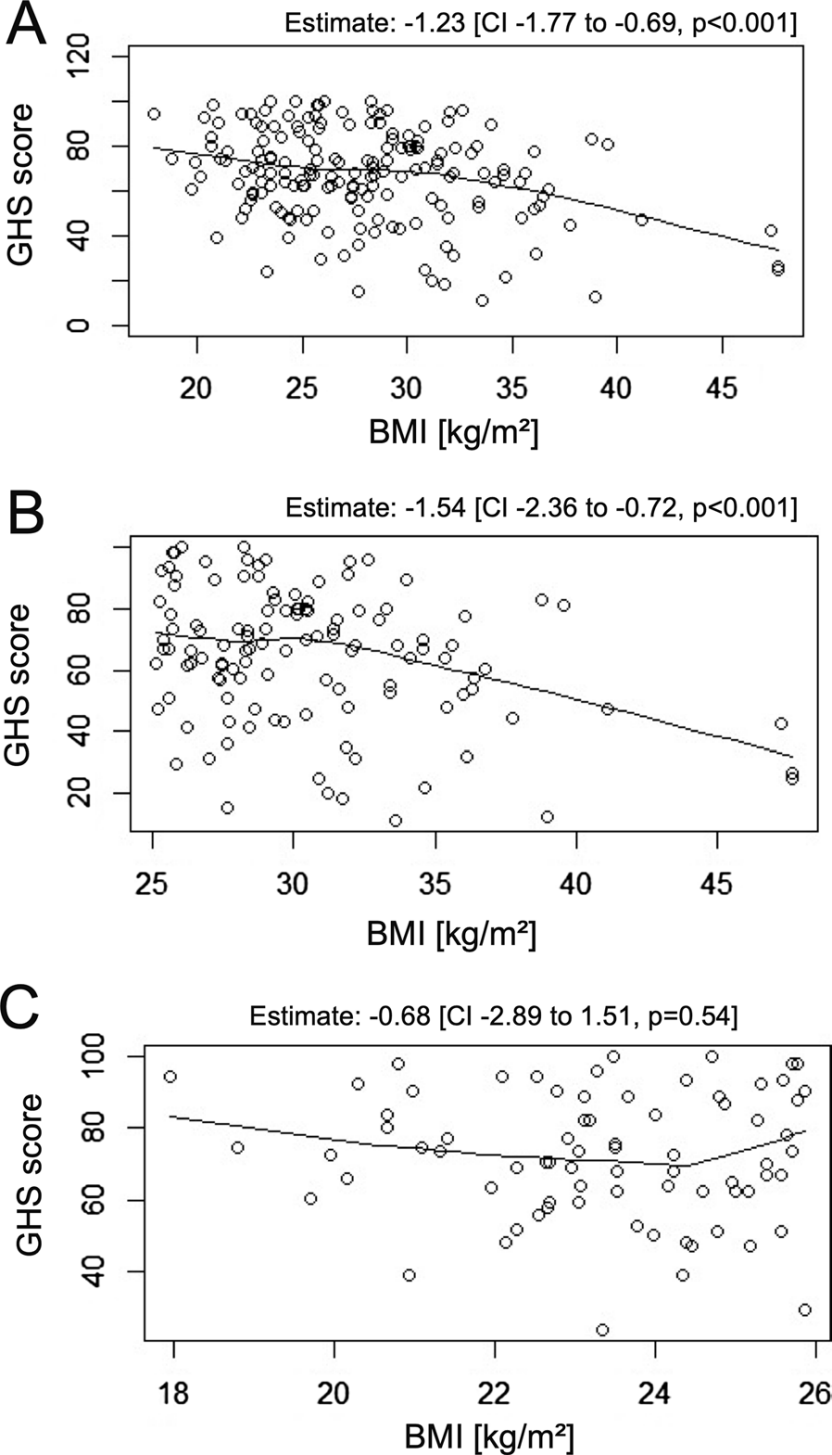
Linear model for BMI and GHS. Linear models were calculated to assess, whether BMI affects GHS in KSQ. **A** BMI inversely influenced GHS with an estimate of − 1.23, meaning that every increase of 1 kg/m^2^ in BMI lead to 1.23 points lower GHS. **B** BMI in sarcoidosis patients with a BMI > 25 kg/m^2^ strongly affects GHS, with an estimate of − 1.54. **C** For patients with BMI < 26 kg/m^2^, no effect of BMI on GHS could be detected

Corresponding to observations for the LUNG score, BMI is the negative driver for GHS (estimate -1.08, 95% CI − 1.83 to − 0.43, *p* < 0.001). Figure 2 shows the univariable model for BMI Overall, there is a modest negative effect of BMI on GHS (estimate − 1.23, *p* < 0.001, Fig. 2A) that is mainly driven by individuals with an elevated BMI (> 25 kg/m^2^) (estimate − 1.54, 95% CI − 2.36 to − 0.72, *p* < 0.001, Fig. 2B). For adipose individuals with a BMI ≥ 30 kg/m^2^, the estimate was − 2.04 points on GHS (*p* = 0.002, data not shown). In contrast, patients with a normal BMI GHS is not influenced by BMI (estimate − 0.68, 95% CI − 2.89 to 1.51, *p* = 0.54, Fig. 2C).

Underlying immunosuppressive therapy (e.g. corticosteroid therapy, Additional file 1: Fig. S4) did not influence BMI, however we noticed an inverse correlation between BMI and MED score with higher BMI correlated with lower MED scores (R = − 0.16, *p* = 0.06). These results may point towards increased worries on (possibly necessary) therapies in patients with higher BMI.

### FeV1 exerts slight influence in GHS

Sarcoidosis affects mainly the lungs and LUNG score in KSQ explained partially GHS as experienced by patients. However, GHS is only slightly influenced by FeV1 in a linear regression model (Table 5; estimate 0.178, 95% CI 0.02 to 0.34, *p* = 0.032), whereas GHS was not influenced by all other lung function parameters. This effect was confirmed in a multivariable model including all lung function parameters. In this model, FeV1 influenced GHS with an estimate of 0.40 (95% CI 0.07 to 0.72, *p* = 0.016). This effect remained robust when adding BMI or serological markers to the model. In detail, only FeV1 (estimate 0.34, 95% CI 0.02 to 0.70, *p* = 0.005) and BMI (estimate − 1.14, 95% CI − 1.80 to − 0.47, *p* < 0.001) directly influenced GHS, whereas all other tested parameters remained without measurable influence on GHS.

**Table 5:** Influence of lung function on GHS in a univariable model with GHS as dependent variable and each lung function parameter as independent variable

	Estimate	CI	*p*-value
FeV1	**0.178**	**0.015 to 0.341**	**0.032**
FVC	0.077	− 0.094 to 0.249	0.376
TLC	0.084	− 0.131 to 0.300	0.439
DLCO	0.135	− 0.061 to 0.331	0.177

Values with statistical significance defined as *p* < 0.05 were indicated in bold

### Serological parameters do not correlate with GHS

**Fig. 3:**
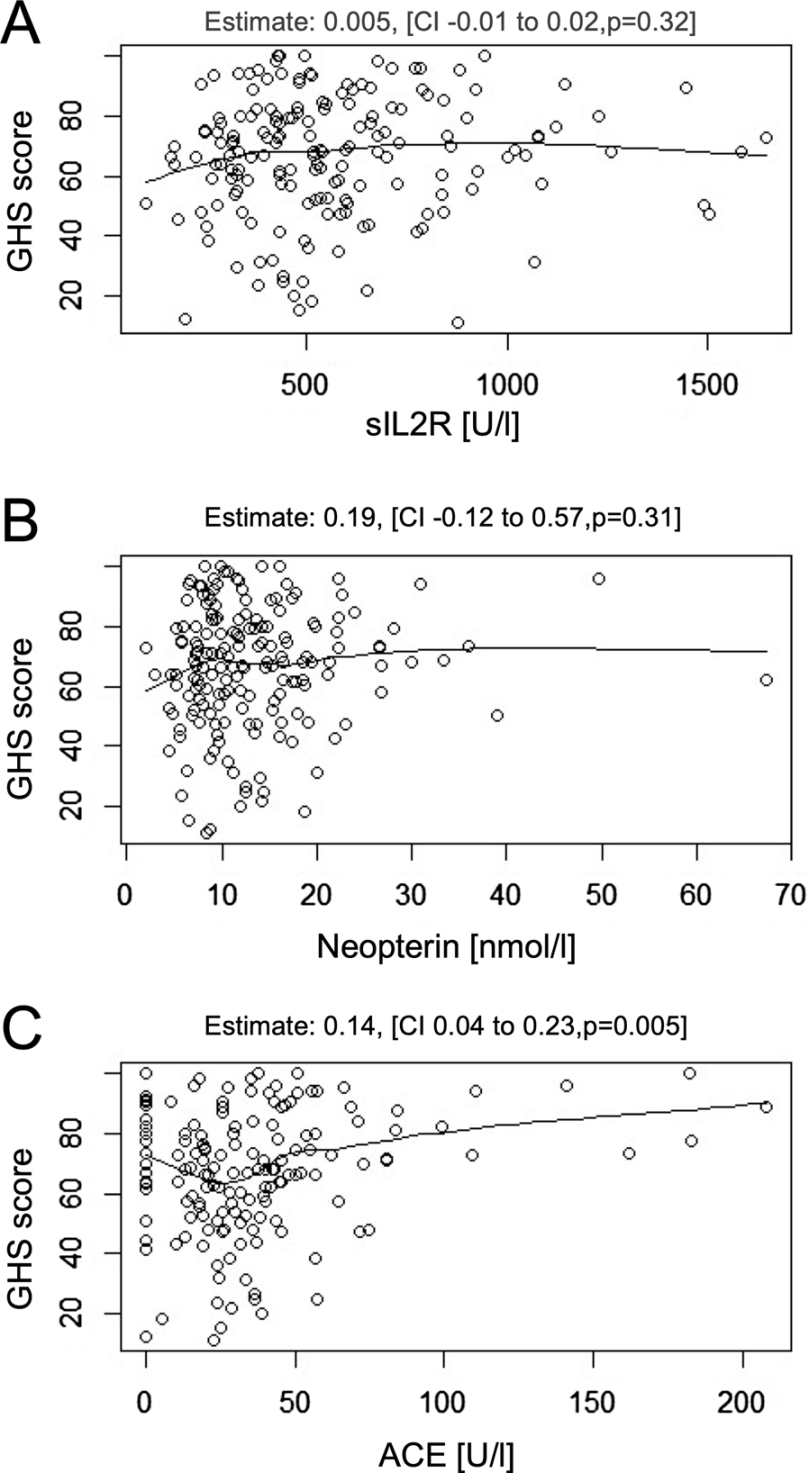
Linear models for serological parameters and their influence on GHS. Linear models were calculated to assess the influence of serological parameters for GHS. **A** sIL2R did not influence GHS in sarcoidosis patients. **B** Neopterin did not influence GHS in sarcoidosis patients. **C** ACE slightly influenced GHS with higher ACE levels leading to better GHS scores

As constitutional complaints in sarcoidosis patients are supposed to derive from inflammation, we tested whether serological parameters of sarcoidosis activity (soluble interleukin-2 receptor, ACE and neopterin) correlate with GHS. In the bivariate correlation analysis according to Pearson, neither sIL2R nor neopterin correlate with GHS (Additional file 1: Fig. S5A and S5B), however we noticed a weak positive correlation between ACE and GHS, which however conflicts with the hypothesis that greater granuloma burden causes lower GHS (Additional file 1: Figure S5C). Similarly, in a linear model we did not observe any correlation of sIL2R or neopterin with GHS (Fig. 3A and B), whereas this linear model suggested a slight effect of ACE levels on GHS, with higher ACE levels resulting in better GHS (Fig. 3C). This effect remained stable for patients independently of the BMI (data not shown).

## Discussion

Sarcoidosis is a granulomatous disease of unknown origin and, beyond its acute presentation, can take a chronic course and thereby may affect virtually every organ while favoring lung involvement [1, 2, 13, 14]. Organ involvement often requires immunosuppressive therapy. In addition to direct organ involvement, constitutional complaints like dyspnea on exertion (DOE), fatigue, pain and weakness may limit patients’ wellbeing even in the absence of direct organ manifestation [15, 16], however, these complaints are difficult to assess and especially to measure e.g. for clinical trials. The King’s Sarcoidosis Questionnaire is a relatively new health status measure to assess the patients’ perspective on their disease [7] and recently was validated for Dutch and German [8, 9]. For this questionnaire, there is a lack of knowledge, how disease assessment by routine follow-up parameters explains KSQ as a surrogate of health perceptation by affected persons.

In this study, we analyzed to which extent follow-up parameters in sarcoidosis can explain KSQ values and whether there are independent and supplementary information obtained by using the KSQ. We therefore analyzed KSQ values obtained in the validation cohort taking clinical data in consideration, focusing on the GHS and the LUNG scores because most of the patients had pulmonary manifestation of sarcoidosis (Additional file 1: Table S1).

There are three main observations. First, the questionnaire adds significant information about the patients’ health status beyond classical parameters obtained in routine follow-up; second, BMI impacts patients’ reported well-being; and third, of all lung function parameters, FeV1 correlates most strongly with KSQ scores, both, for female and male..

In our cohort, GHS adds information of patients’ health perceptation beyond other clinical parameters assessed in routine follow-up. As could be expected, GHS is influenced by the subdomain scores, which were generally lower in patients with respective organ manifestation or drug therapy. However, the influence of each subdomain or any organ manifestation only partially explained GHS and organ manifestations did not fully explain respective subdomain scores either. Importantly, single organ involvement did not influence GHS apart from bone involvement (Table 4), which can be considered as a surrogate of multiple organ involvement [17] affecting GHS (Additional file 1: Table S4). Additionally, we did not observe that serological parameters correlate with GHS or LUNG scores (Table 3 and Fig. 3) besides ACE, which slightly associated with GHS score (Fig. 3C). This is noteworthy, because reduced quality of life of sarcoidosis is often hypothesized to relate to inflammatory activity. The effect of ACE in this context is against the expectations, as higher ACE values signify higher GHS scores. However, one has to consider that ACE values were not genotype-corrected [18–20] and were the most missing data in the cohort, because they were not routinely measured since there is no generally accepted standardization of the test. Inflammatory parameters from bronchoalveolar lavage and peripheral blood gauge pathological mechanism and may reflect inflammatory activity of sarcoidosis, allowing identification of patients at risk for progression and therapeutic need [21–26]. Most interestingly, these mechanisms and inflammatory activity do not hamper general health as shown in Fig. 3 and Additional file 1: Figure S5.

Patel et al. [7] demonstrated that lung function parameters influence LUNG score, which we could reproduce in our cohort. FVC, FeV1, TLC and DLCO all correlate with LUNG score (Fig. 1). Of note, correlation differs between male and female and was stronger for the latter one. We did not observe any other difference between male and female in our cohort, neither for questionnaire score nor for clinical, serological or lung function parameter.

In contrast to the findings from Patel et al. [7], FeV1 and DLCO were the most important drivers for LUNG score in our cohort (Additional file 1: Table S3). Their effect was still detectable in multivariate models integrating serological parameters, age, sex, BMI, and radiological type (Table 3). Notably, VIF below 10 indicates that multicollinearity is not mainly causative of these findings [12]. VIF values 5.85 and 4.42 for FVC and FeV1 respectively can be easily anticipated by the fact that in restrictive lung diseases FeV1 depends on FVC. However, the importance of FeV1 for LUNG and GHS score is an interesting observation in regard of a recent description of different ventilatory defects in a large cohort of sarcoidosis patients [27]; an obstructive ventilator defect was found in approximately 15% of patients in our cohort. Especially in obstructive and mixed ventilatory defects, FeV1 may better reflect airway involvement and thereby explain reduced quality of life. The importance of FeV1 for patients’ perception of sarcoidosis-associated well-beings further emphasized by the fact that FeV1 remained the only significant lung function parameter that influenced GHS in the univariable linear regression analysis (Table 5). This effect remained robust after including serological, radiological or clinical parameters as independent variables. Considering a minimal clinical important difference for the GHS domain of 8 and the LUNG domain of 4 [28], an absolute increase or decrease in FeV1 of 6% or 12% will result in better or worse quality of life as assessed by the LUNG or GHS score, respectively.

The third important point of the analysis is the role of BMI for patients’ quality of life. Our analysis of the cohort does not allow to conclude, whether obesity is a reason for or consequence of reduced quality of life, however obesity has been recognized as prevalent in sarcoidosis patients [29] and affecting quality of life [30, 31]. Vice versa reduced quality of life may result in inactivity and obesity resulting in a vicious circle. Obesity additionally affects lung function parameters and sensation of dyspnea [32, 33], but effect of BMI on LUNG and GHS score was independent of lung function impairment (Tables 3 and 4) and remains robust in all analyses. We did not observe differences in BMI between patients with and without immunosuppressive therapy (Additional file 1: Fig. S4), but our analysis could not rule out that obesity resulted from previous corticosteroid therapy with its immanent side effect of weight gain [34]. In this context one may interpret the observation that worries about medication correlate with BMI (R = − 0.16, *p* = 0.06), demonstrating an interplay between weight and concerns about drug therapy. The robust influence of obesity on sarcoidosis-associated quality of life is noteworthy in the context that several data support an influence of obesity on the inflammatory environment [35–37] which may propagate autoimmune diseases like sarcoidosis [38, 39]. In this context, one might speculate whether life style modifications like diet may alter the inflammatory milieu and patients’ reported quality of life [40, 41].

In summary, our study shows that GHS gauges an aspect of patients’ suffering from sarcoidosis, which is not captured by the clinical parameters in use and is of relevance for patients monitoring and clinical decisionmaking.

There are several limitations of this study. First, most of the patients included in the study were recruited in a tertiary pneumological center, which may bias the cohort towards more severely and chronically diseased patients. However, especially in this cohort of patients, the use of questionnaires to assess patients’ health status and to adapt therapy is especially useful, whereas its role in patients presenting with acute or uncomplicated sarcoidosis is debatable. Second, KSQ values were determined at a single time point, which does not allow answering the question about its value in therapeutic decisions. A recent publication analysed the KSQ score in the follow-up of patients underscoring its use in patient care [28]. Third, the application of multivariable linear models to assess factors that influence patient-reported outcomes represent an exploratory and artificial mathematical approach, that leaves out a certain amount of unmeasurable information. Accordingly, the adjusted r-squares in our analysis only demonstrated a moderate fitting of the applied models, which was confirmed in cross-validation strategies. Nevertheless, the effect of BMI and FeV1 remained robust over all multivariable models. However, as we outlined before, clinical data only explain partially patient-reported health-related quality of life.

## Conclusions

Even with these limitations in mind, the study contributes some important insights. Most importantly, we could demonstrate that KSQ adds important additional information to routinely monitored parameters in sarcoidosis care. Therefore, the questionnaire may represent a tool in patient care and also a relevant instrument for clinical studies in sarcoidosis. Furthermore, our data suggest an important role of FeV1 at least for the patients’ self-perception of well-being. Interestingly, there seems to be a slight difference in the perception between male and female, as female LUNG scores correlate better with lung function compared to male. Lastly, the role of BMI in sarcoidosis and associated quality of life is emphasized by our study and raises the question, whether addressing body weight may be a treatable trait for sarcoidosis-associated constitutional complaints.

## Supplementary Information

**Additional file 1: Table S1.** Organ involvement of the study cohort. **Table S2**. Multivariable model for the influence of KSQ subdomains on KSQ GHS domain (adjusted R² = 0.56, p<0.001). **Table S3** Univariable model with LUNG as dependent variable and each lung function parameter as independent variable. **Table S4** Univariable model with GHS as dependent variable and organ involvement as independent variable. **Figure S1**. GHS score for female and male. **Figure S2** Organ-specific scores of the KSQ in sarcoidosis patients with and without affected organ. **Figure S3**. Influence of subdomains on GHS score. **Figure S4**. Correlation between BMI and drug therapy demonstrating that BMI was not influenced by drug therapy. **Figure S5**. Correlation between serological parameters and GHS

## Data Availability

Anonymized data and statistical codes can be obtained from the corresponding author upon reasonable request and consideration of regulatory and legal requirements.

## Abbreviations

ACE: Angiotensin-converting enzyme
BMI: Body mass index
DLCO: Diffusion Capacity of the Lungs for Carbon Monoxide
E(YE): Eye domain of the KSQ
FeV1: Forced expiratory volume in 1 s
FVC: Forced vital capacity
GHS: General Heath Status
KSQ: King’s Sarcoidosis Questionnaire
L(UNG): Lung score of the KSQ
M(ED): Medication domain of the KSQ
QoL: Quality of life
sIL2R: Soluble interleukin-2 receptor
S(KIN): Skin score of the KSQ
TLC: Total lung capacity
