# Supplementary material for: FeV1 and BMI influence King’s Sarcoidosis Questionnaire score in sarcoidosis patients
Source: BMC Pulm Med. 2021 Dec 3;21:395. doi: 10.1186/s12890-021-01761-7 (PMC8643005; doi:10.1186/s12890-021-01761-7)
Supplement: Supplementary file 1 — Additional file 1: Table S1. Organ involvement of the study cohort. Table S2. Multivariable model for the influence of KSQ subdomains on KSQ GHS domain (adjusted R² = 0.56, p<0.001). Table S3 Univariable model with LUNG as dependent variable and each lung function parameter as independent variable. Table S4 Univariable model with GHS as dependent variable and organ involvement as independent variable. Figure S1. GHS score for female and male. Figure S2 Organ-specific scores of the KSQ in sarcoidosis patients with and without affected organ. Figure S3. Influence of subdomains on GHS score. Figure S4. Correlation between BMI and drug therapy demonstrating that BMI was not influenced by drug therapy. Figure S5. Correlation between serological parameters and GHS [file 12890_2021_1761_MOESM1_ESM.docx]

**Supplementary Material**

**Supplementary Tables:**

**Supplementary Table S1:** Organ involvement of the study cohort.

| **Organ** | **All [%]** | **Female (f) [%]** | **Male (m) [%]** | **p (f vs. m)** |
| --- | --- | --- | --- | --- |
| **Lung** | 87.85 | 87.34 | 88.24 | 1.0 |
| **Lymph nodes** | 62.98 | 65.82 | 60.78 | 0.59 |
| **Arthritis** | 3.31 | 5.06 | 1.96 | 0.41 |
| **Liver** | *6.63* | *2.53* | *9.80* | *0.07* |
| **Spleen** | 4.97 | 3.80 | 5.88 | 0.73 |
| **Kidney** | 6.08 | 5.06 | 6.86 | 0.76 |
| **Heart** | 3.31 | 3.80 | 2.94 | 1.0 |
| **Bones** | 4.42 | 5.06 | 3.92 | 0.73 |
| **Skin** | 12.71 | 11.39 | 13.73 | 0.81 |
| **Eyes** | 9.39 | 6.33 | 11.76 | 0.30 |
| **central nervous system** | 7.73 | 5.06 | 9.80 | 0.27 |
| **Therapy** | 53.89 | 54.17 | 53.68 | 1.0 |

**Supplementary Table S2:** Multivariable model for the influence of KSQ subdomains on KSQ GHS domain (adjusted R² = 0.56, p<0.001).

| **Subdomain** | **Estimated effect on GHS** | **CI** | **p-value** | **VIF** |
| --- | --- | --- | --- | --- |
| LUNG | **0.45** | **0.34 to 0.55** | **<0.001** | 1.36 |
| SKIN | **0.11** | **0.02 to 0.20** | **0.017** | 1.15 |
| EYE | **0.11** | **0.02 to 0.21** | **0.015** | 1.22 |
| MED | **0.16** | **0.08 to 0.24** | **<0.001** | 1.27 |

**Supplementary Table S3:** Univariable model with LUNG as dependent variable and each lung function parameter as independent variable.

| **parameter** | **Estimate on LUNG** | **CI** | **p-value** |
| --- | --- | --- | --- |
| FVC | **0.30** | **0.13 to 0.47** | **<0.001** |
| FeV1 | **0.43** | **0.27 to 0.59** | **<0.001** |
| TLC | **0.32** | **0.09 to 0.56** | **0.006** |
| DLCO | **0.53** | **0.13 to 0.71** | **0.001** |

**Supplementary Table 4:** Univariable model with GHS as dependent variable and organ involvement as independent variable.

| **Organ involved** | **Estimate on GHS** | **CI** | **p-value** |
| --- | --- | --- | --- |
| Lung only | 1.4 | -6.71 to 9.53 | 0.732 |
| Lung + 1 organ | 1.2 | -5.19 to 7.62 | 0.709 |
| Lung + 2 organs | 1.8 | -5.83 to 9.52 | 0.630 |
| Lung + 3 organs | -1.64 | -17.41 to 14.13 | 0.837 |
| Lung + 4 organs | **-41.635** | **-57.38 to -25.89** | **< 0.001** |

**Supplementary Figures:**

**
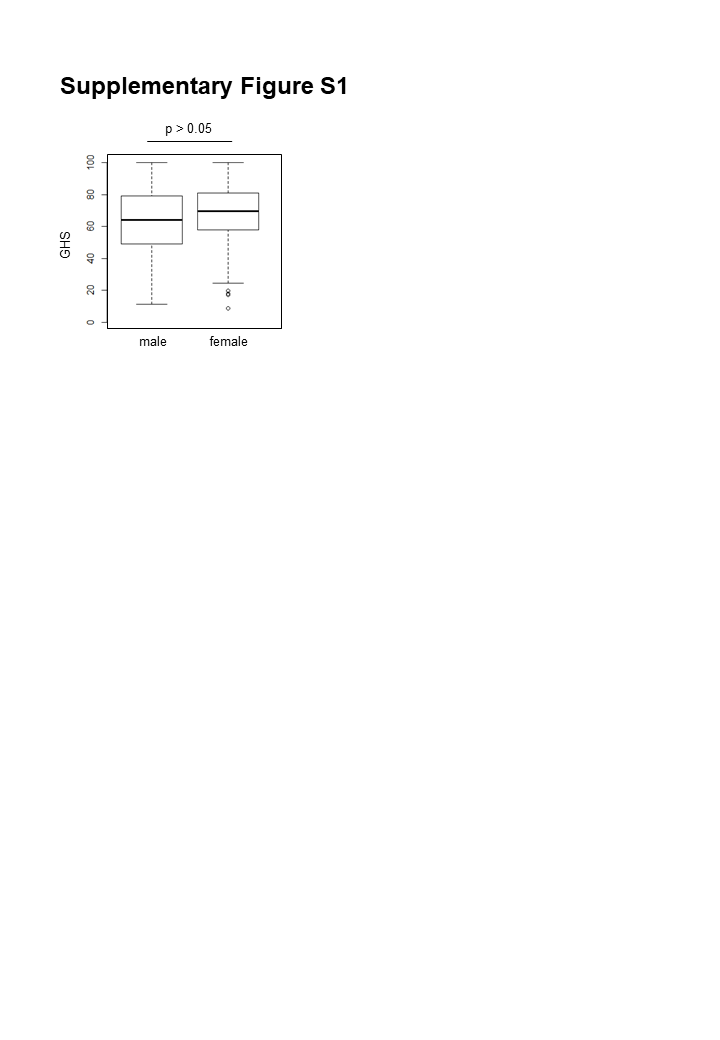
**

**Supplementary Figure S1:** GHS score for female and male.

GHS score was compared between female and male and no statistically significant difference could be observed.

**
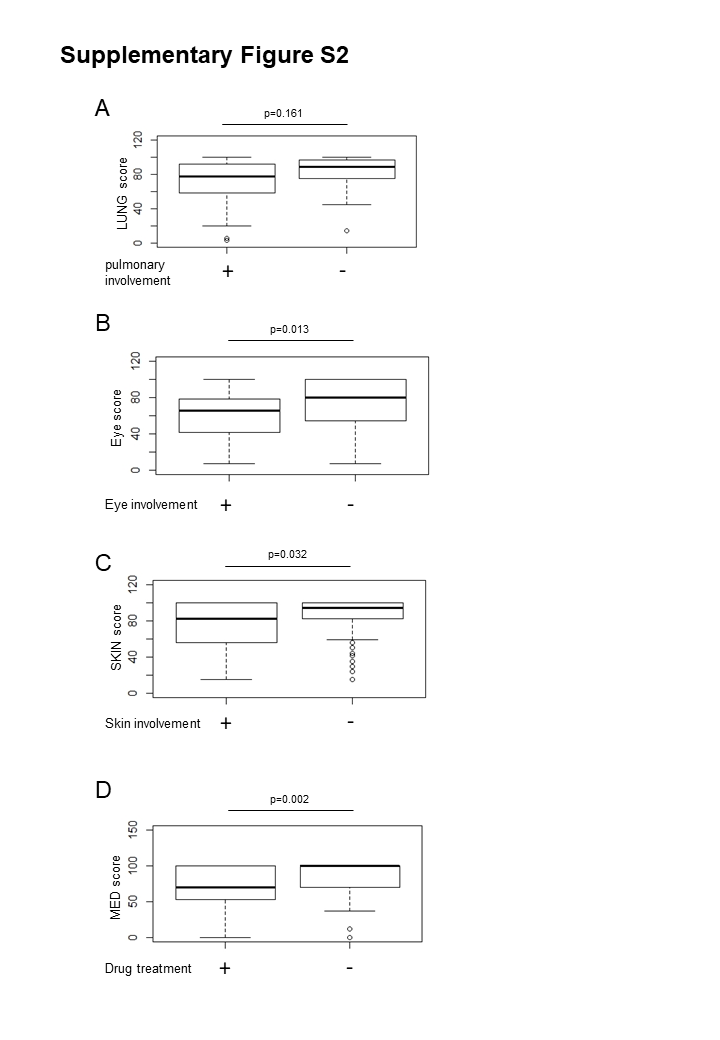
**

**Supplementary Figure S2:** Organ-specific scores of the KSQ in sarcoidosis patients with and without affected organ.

Subdomain scores of GHS were compared between patients with and without respective involvement.

1. Patients with (+) or without (-) lung involvement indicate lower LUNG scores in the KSQ, however difference was not statistically significant.
2. Sarcoidosis patients with (+) or without (-) eye involvement indicate lower EYE score in KSQ compared to patients without eye involvement.
3. Sarcoidosis patients with (+) or without (-) skin involvement indicate lower SKIN score in KSQ compared to patients without skin involvement.
4. Patients with (+) or without (-) current drug therapy for sarcoidosis indicate lower MED score in KSQ compared to patients without current drug therapy.

**
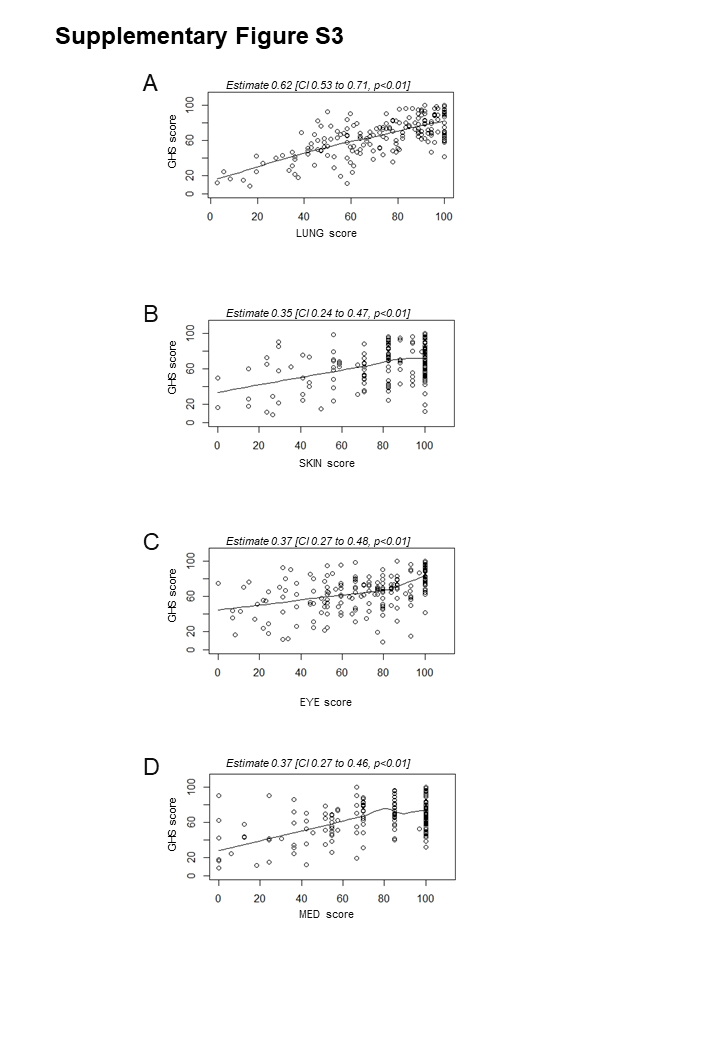
**

**Supplementary Figure S3:** Influence of subdomains on GHS score. Univariate linear models were calculated to assess the influence of subdomain scores on GHS.

1. LUNG score significantly influenced GHS score with an estimate of 0.62.
2. SKIN score significantly influenced GHS score with an estimate of 0.35.
3. EYE score significantly influenced GHS score with an estimate of 0.37.
4. DRUG score significantly influenced GHS score with an estimate of 0.37.


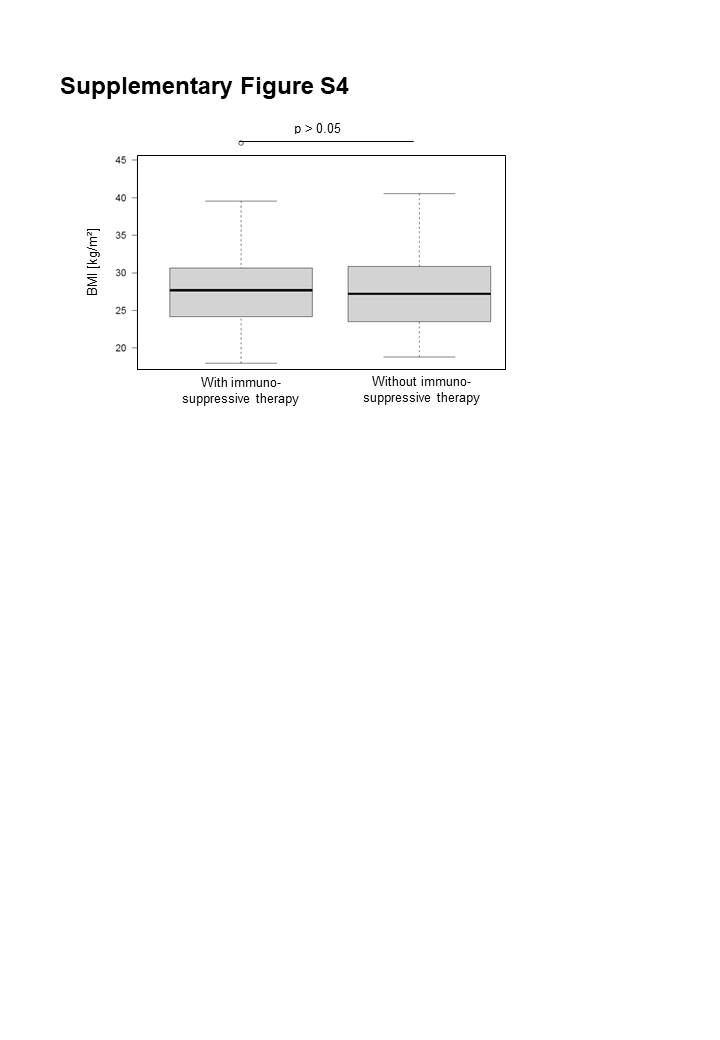


**Supplementary Figure S4:** Correlation between BMI and drug therapy demonstrating that BMI was not influenced by drug therapy.


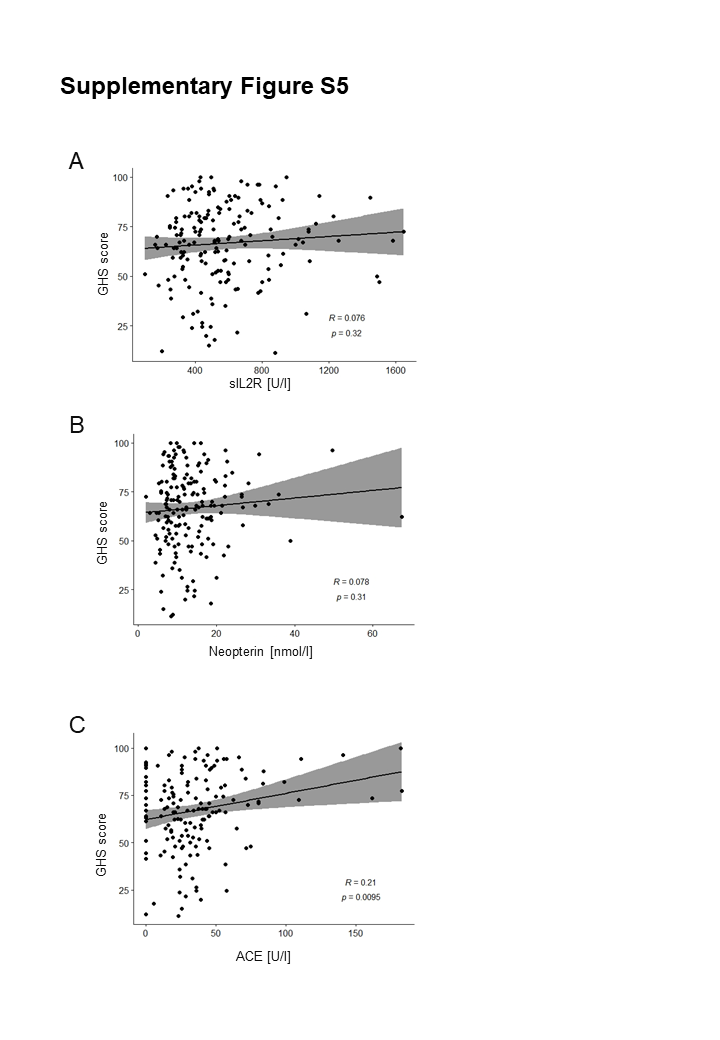


**Supplementary Figure S5:** Correlation between serological parameters and GHS

Pearson’s correlations were calculated for serological parameters and GHS.

1. sIL2R did not correlate with GHS in sarcoidosis patients.
2. Neopterin did not correlate with GHS in sarcoidosis patients.
3. ACE slightly correlated with GHS.
